# Stratified exercise therapy compared with usual care by physical therapists in patients with knee osteoarthritis: A randomized controlled trial protocol (OCTOPuS study)

**DOI:** 10.1002/pri.1819

**Published:** 2019-11-28

**Authors:** Jesper Knoop, Joost Dekker, Marike van der Leeden, Mariëtte de Rooij, Wilfred F.H. Peter, Leti van Bodegom‐Vos, Johanna M. van Dongen, Nique Lopuhäa, Kim L. Bennell, Willem F. Lems, Martin van der Esch, Thea P.M. Vliet Vlieland, Raymond W.J.G. Ostelo

**Affiliations:** ^1^ Department of Health Sciences Vrije Universiteit Amsterdam Amsterdam The Netherlands; ^2^ VU University Medical Center, Department of Rehabilitation Medicine Amsterdam UMC Amsterdam The Netherlands; ^3^ Amsterdam Rehabilitation Research Centre Reade Amsterdam The Netherlands; ^4^ Department of Orthopaedics Leiden University Medical Center (LUMC) Leiden The Netherlands; ^5^ ReumaNederland Amsterdam The Netherlands; ^6^ School of Health Sciences, Department of Physiotherapy University of Melbourne Melbourne Victoria Australia; ^7^ Amsterdam University of Applied Sciences Amsterdam The Netherlands

**Keywords:** cluster randomized controlled trial, exercise therapy, knee osteoarthritis, stratified care

## Abstract

**Objectives:**

Knee osteoarthritis (OA) is characterized by its heterogeneity, with large differences in clinical characteristics between patients. Therefore, a stratified approach to exercise therapy, whereby patients are allocated to homogeneous subgroups and receive a stratified, subgroup‐specific intervention, can be expected to optimize current clinical effects. Recently, we developed and pilot tested a model of stratified exercise therapy based on clinically relevant subgroups of knee OA patients that we previously identified. Based on the promising results, it is timely to evaluate the (cost‐)effectiveness of stratified exercise therapy compared with usual, “nonstratified” exercise therapy.

**Methods:**

A pragmatic cluster randomized controlled trial including economic and process evaluation, comparing stratified exercise therapy with usual care by physical therapists (PTs) in primary care, in a total of 408 patients with clinically diagnosed knee OA. Eligible physical therapy practices are randomized in a 1:2 ratio to provide the experimental (in 204 patients) or control intervention (in 204 patients), respectively. The experimental intervention is a model of stratified exercise therapy consisting of (a) a stratification algorithm that allocates patients to a “high muscle strength subgroup,” “low muscle strength subgroup,” or “obesity subgroup” and (b) subgroup‐specific, protocolized exercise therapy (with an additional dietary intervention from a dietician for the obesity subgroup only). The control intervention will be usual best practice by PTs (i.e., nonstratified exercise therapy). Our primary outcome measures are knee pain severity (Numeric Rating Scale) and physical functioning (Knee Injury and Osteoarthritis Outcome Score subscale daily living). Measurements will be performed at baseline, 3‐month (primary endpoint), 6‐month (questionnaires only), and 12‐month follow‐up, with an additional cost questionnaire at 9 months. Intention‐to‐treat, multilevel, regression analysis comparing stratified versus usual care will be performed.

**Conclusion:**

This study will demonstrate whether stratified care provided by primary care PTs is effective and cost‐effective compared with usual best practice from PTs.

## 1 INTRODUCTION

Osteoarthritis (OA) is one of the most common chronic health conditions and a leading cause of pain and disability among adults (Allen & Golightly, 2015). Knee OA is also a highly heterogeneous disease, with large differences between patients in disease onset and course, symptoms, and treatment response (Bijlsma, Berenbaum, & Lafeber, 2011; Felson, 2010). Exercise therapy is recommended as a first‐step treatment for knee OA, next to pain medication and diet (Hochberg et al., 2012; Zhang et al., 2010). If this conservative treatment fails, knee replacement surgery can be considered. There is strong, high‐quality evidence for the effectiveness of exercise therapy on knee pain and physical function, compared with no exercise therapy, with similar or even larger effects than pain medication or any other conservative treatment (Fransen et al., 2015). These effects have been found not only in mild OA but also in severe, end‐stage OA (Knoop et al., 2014; Skou et al., 2018). Recent evidence also suggests that the beneficial effects of exercise therapy are primarily driven by improvements in upper leg muscle strength (Knoop et al., 2015; Runhaar, Luijsterburg, Dekker, & Bierma‐Zeinstra, 2015), highlighting the important role of strength training for those patients with muscle weakness. On the other hand, for patients with sufficiently strong muscles, muscle strengthening is not likely to result in improvements in pain and physical function (Edelaar et al., 2017). Therefore, it is plausible that for these patients, only a minimal intervention focusing on self‐management strategies (including advices how to stay physically active, without “overloading” the affected knee joint) rather than muscle strengthening should be provided, thereby avoiding unnecessary care.

Although effective, the average effect sizes of exercise therapy on knee pain and physical function are still modest (i.e., between‐group effect size of 0.5 for both pain and physical function, compared with “no treatment”; mean improvement [within‐group] of 27% [pain] and 26% [physical function], Fransen et al., 2015). This may be attributed to a “one‐size‐fits‐all” approach. Instead, given the large heterogeneity of knee OA, a stratified approach to exercise therapy may be superior. The effectiveness and cost‐effectiveness of stratified exercise therapy has been proven in patients with low back pain (Hill et al., 2011) but has not been tested in other patient groups. To our knowledge, no model of stratified exercise therapy has yet been developed for patients with knee OA. Recently, we were able to identify five clinically relevant phenotypes (subgroups) of knee OA patients based on easily obtainable patient characteristics (i.e., radiographic severity, body mass index [BMI], upper leg muscle strength, and depressive mood) and replicated this finding in a second cohort study (Knoop et al., 2011; van der Esch et al., 2015). This replication indicated that these five phenotypes can be considered valid and clinically relevant. Therefore, we developed a phenotype‐based model of stratified exercise therapy. We decided to combine two identified phenotypes that only differed in radiographic severity, as we previously demonstrated that effects of exercise therapy are independent of knee joint damage severity (Knoop et al., 2014). This resulted in a model in which patients were allocated into a “low muscle strength subgroup,” “high muscle strength subgroup,” “obesity subgroup,” or “depressive phenotype,” which all receive subgroup‐specific exercise therapy.

A pilot study (Knoop et al., 2019), testing this model of stratified care in the primary care setting (*n* = 50), provided promising results and thereby impetus for a fully powered trial. The pilot study demonstrated clinical improvements on knee pain (within‐group effect size of 1.1 and mean improvement of 37%) and physical function (0.7 and 20%). In addition, participating PTs provided on average less treatment sessions compared with usual care (10 vs. 17 sessions, Leemrijse et al., 2016). Finally, in interviews, PTs were positive about the applicability of the stratification tool (i.e., easy to use and not time‐consuming), and both patients (i.e., easy‐to‐perform exercises and tailored patient education) and PTs (i.e., subgroup‐specific treatment protocols) acknowledged the added value of the stratified care model. The pilot study also revealed some limitations in the model, and as such, we adapted our model to address this, prior to our planned trial. First, because one of the four subgroups (“depression subgroup”) had a very low prevalence, we decided to remove this subgroup from our model, which we discussed more in detail in our pilot study manuscript (Knoop et al., 2019). Second, clinical improvements in the obesity subgroup were lower compared with the other subgroups. Therefore, we decided to optimize our intervention of this subgroup by adding an obligatory weight‐loss intervention from a dietician, instead of only advising the patient to consult a dietician. This combined intervention of weight loss and exercise therapy can be expected to be more effective in this subgroup, based on recent evidence of superior effects in reducing clinical symptoms over weight loss only or exercise only in overweight and obese patients with knee OA (Messier et al., 2013). Third, we decided to reduce the cut‐off point for the obesity subgroup from BMI of 35 to 30, so every person with obesity (i.e., BMI ≥ 30) receives this combined, interdisciplinary intervention. Fourth and final, in our pilot study, knee extension strength was measured by a hand‐held dynamometer, which is a costly instrument demonstrating large variations. Therefore, we decided to replace this instrument into the 30‐s chair stand test, which is a reliable and easy‐to‐perform, functional test of lower body strength (Dobson et al., 2012).

We hypothesize that stratified exercise therapy by PTs, according to our (adapted) model of stratified care with three subgroups (with an additional dietary intervention for one subgroup only), will be effective in reducing knee pain and improving physical functioning and cost‐effective for quality‐adjusted life years in patients with knee OA, compared with usual, “nonstratified” exercise therapy by PTs. We are currently testing this hypothesis in a cluster randomized controlled trial (CRCT; OCTOPuS study, acronym for Optimization of exerCise Therapy in patients with knee Osteoarthritis in a Primary care Setting).

## 2 METHODS

### 2.1 Trial design

We are performing a pragmatic, parallel‐group CRCT, comparing the effectiveness of stratified exercise therapy by PTs (experimental intervention) with usual, nonstratified care by PTs (control intervention), with additional economic and (both quantitative and qualitative) process evaluation. This study is performed in a primary care setting, for which PT practices have been recruited and randomized to either the experimental or control intervention. The study period is 12 months, with measurements at baseline (t0), 3‐month (t3; primary end point), 6‐month (t6), 9‐month (t9), and 12‐month follow‐up (t12).

### 2.2 Participants

PTs screen for eligibility in new patients presenting to them with persisting knee pain, referred by either their general practitioner or orthopaedic surgeon, or consulted the PT directly. Patients should meet all of the following inclusion criteria to be eligible:

- knee pain persisting for at least 3 months with pain severity during walking ≥2/10 on a Numeric Rating Scale (checked during history taking by PT);
- clinical diagnosis of knee OA according to the criteria of the American College of Rheumatology (Altman et al., 1986; checked during physical examination by PT); and
- providing informed consent (checked by both PT and researcher).

Patients are excluded if any of the following exclusion criteria are met (which are all checked during history taking by PT):

- age <40 or >85 years;
- severe knee pain (i.e., Numeric Rating Scale pain severity during walking ≥9/10);
- physical or mental co‐morbidity severely affecting daily life and a contraindication to provide exercise therapy;
- suspicion of chronic widespread pain (i.e., pain present for at least 3 months in at least three joints including left and right side of the body, above and below the waist, and the axial skeleton);
- presence of total knee arthroplasty or on waiting list for total knee arthroplasty in any knee;
- other reasons for knee pain than knee OA (e.g., rheumatoid arthritis and gout);
- received PT in past 6 months because of knee pain;
- received intraarticular injections in past 6 months because of knee pain; and
- insufficient comprehension of Dutch language.

### 2.3 Study procedures

Between January 2019 and February 2020, PTs screen patients for their eligibility during the first consult. During this first consult and at 3‐month follow‐up, PTs also supervise three physical tests (i.e., upper leg muscle strength, BMI, and waist circumference). If a patient is eligible, the PT provides a patient information letter and informed consent form to the patient. In those patients providing informed consent, the researcher collects baseline data by questionnaires. If a patient in the experimental group is allocated to the obesity subgroup, the researcher informs the dietician to start the dietary intervention as soon as possible. This additional dietary intervention is obligatory for patients from this obesity subgroup.

PTs and dieticians register process parameters for each session, including type of interventions applied, side effects, and (for PTs only) training intensity. Follow‐up questionnaires are obtained by the researcher at 3‐, 6‐, 9‐, and 12‐month follow‐up. In these questionnaires, participants also report their adherence to home exercises/physical activities. The researchers continuously record inclusion and dropout rates, as well as (serious) adverse events. In the final phase, the researchers also conduct interviews in a random sample of patients, PTs, and dieticians to collect qualitative information on the content, fidelity, and outcome of the treatment.

### 2.4 Blinding

In order to avoid contamination, patients are blinded for treatment allocation, which is in line with current recommendations for pragmatic CRCTs (Weijer et al., 2011). This means that patients from each treatment arm are informed about their own intervention only, without providing information on the intervention from the other treatment arm. Due to the nature of the intervention, blinding of participating PTs or dieticians is not possible. However, PTs from the control group are not informed about the experimental intervention. All participating PTs and dieticians agreed with the instruction to provide treatment strictly according to the protocol (experimental group) or according to their usual care (control group).

Blinding of the researchers (main researcher and research assistant(s), who are responsible for study coordination, contact with eligible candidates, participants, PTs, and dieticians, and data collection [all questionnaires and t12 physical tests], data monitoring, and data analysis) is not possible, as they need information about treatment allocation for adequate study coordination. However, a researcher blinded for treatment allocation performed the randomization of PT practices and will prepare the dataset and primary statistical analyses. For randomization, we used a web‐based randomization program, with concealment of randomization guaranteed.

### 2.5 Eligibility, allocation, and training of PTs

**Figure 1:**
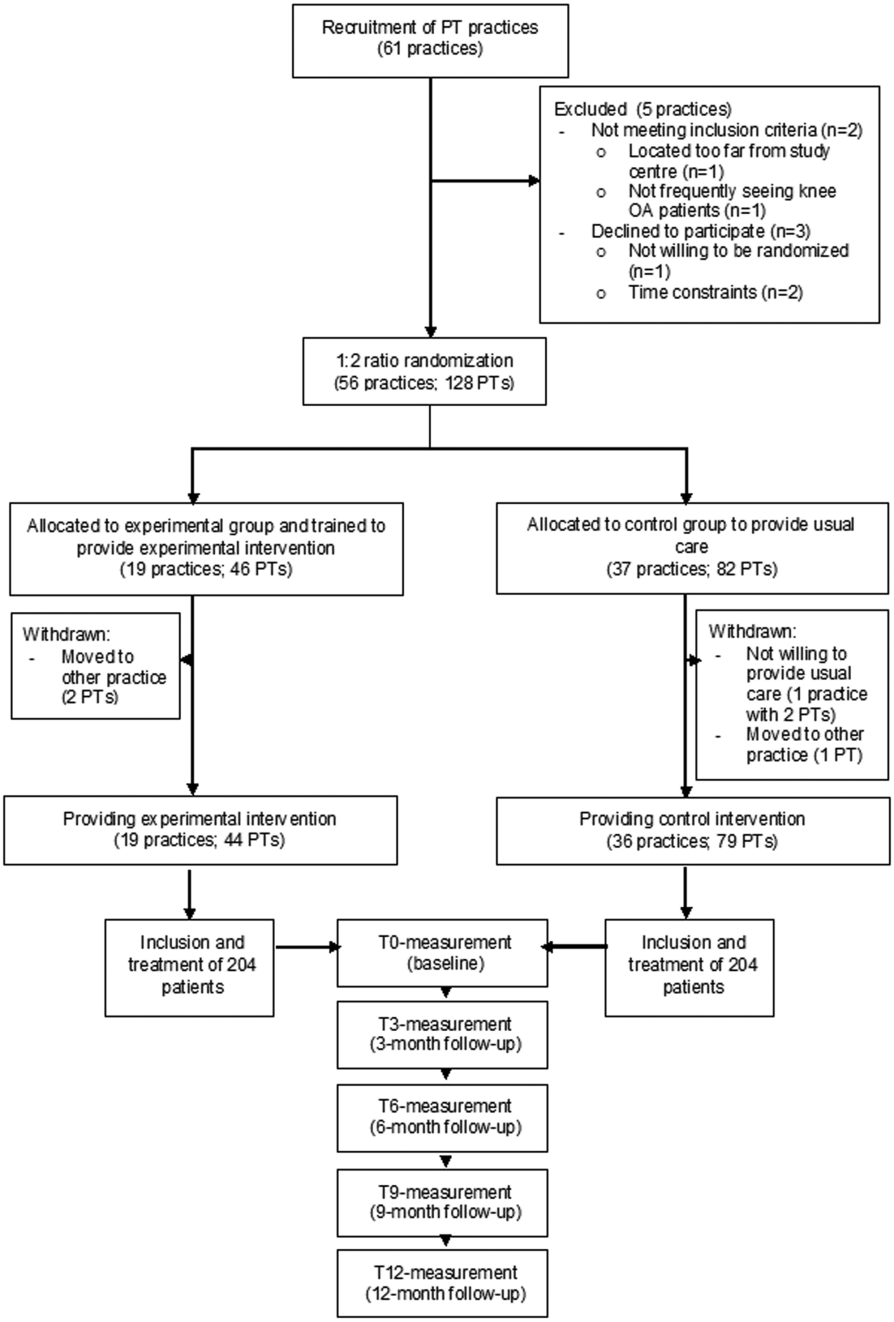
Flow chart of participating physiotherapists (PTs) and patients in the OCTOPuS‐study

PTs were eligible to participate if at least two PTs from their practice can participate, they work in a primary care practice near one of either study centres (*x* and *y*), have facilities to provide exercise therapy, see at least one new knee OA patient per month on average, and were willing to be randomized for this study. This recruitment resulted in 55 eligible PT practices with 128 PTs (with number of PTs per practice varying between two and five; see Figure 1).

Eligible PT practices were randomly allocated to experimental or control group in a 1:2 ratio (i.e., around one third to the experimental group and two thirds to the control group, with each group including 204 patients). The rationale for this allocation ratio is that PTs in the control group will only need to include and treat half of the patients compared with PTs in the experimental group. Because PTs expressed interest in our study primarily because of the training on stratified exercise therapy, a low number of patients will enhance their motivation for this study and minimize the risk of withdrawal. The random allocation was performed separately for practices with 2–3 participating PTs (Stratum 1) and for practices with 4–5 participating PTs (Stratum 2), in order to minimize the risk of not having a 1:2 ratio of PTs allocated to the two treatment arms. Within each stratum, all PT practices were grouped into three, of which one was randomly allocated to the experimental arm and two to the control arm.

PTs allocated to the experimental group were trained to provide the experimental intervention (i.e., stratified exercise therapy; see Section 2.6). A 4‐hr training session was organized to train PTs to provide treatment according to the model of stratified exercise therapy (see Section 2.6), to perform screening, and to supervise physical tests. PTs in the experimental group additionally followed an e‐learning course on the most recent Dutch PT guideline on knee and hip OA, prior to the training session. PTs allocated to the control group were provided a 2‐hr training session to instruct them to provide usual care (see Section 2.6), to perform screening, and to supervise physical tests. The training session on the experimental intervention and the e‐learning course will also be provided to the PTs from the control group but after the study period.

Adherence to the treatment protocols and measurements by the PTs are monitored through regular site visits by the researcher. PTs from the experimental arm can review video‐recorded presentations from the training sessions at any time during the study period. For each participating PT practice randomized to the experimental group, a dietician who routinely collaborates with this PT practice was recruited as well. Dieticians were instructed to deliver a dietary intervention according to the current guideline (van Binsbergen et al., 2010), in patients from the obesity subgroup (see Section 2.6).

### 2.6 Interventions

#### 2.6.1 Experimental intervention

In the experimental group, PTs were trained to provide treatment according to the model of stratified exercise therapy, which consists of

**Figure 2:**
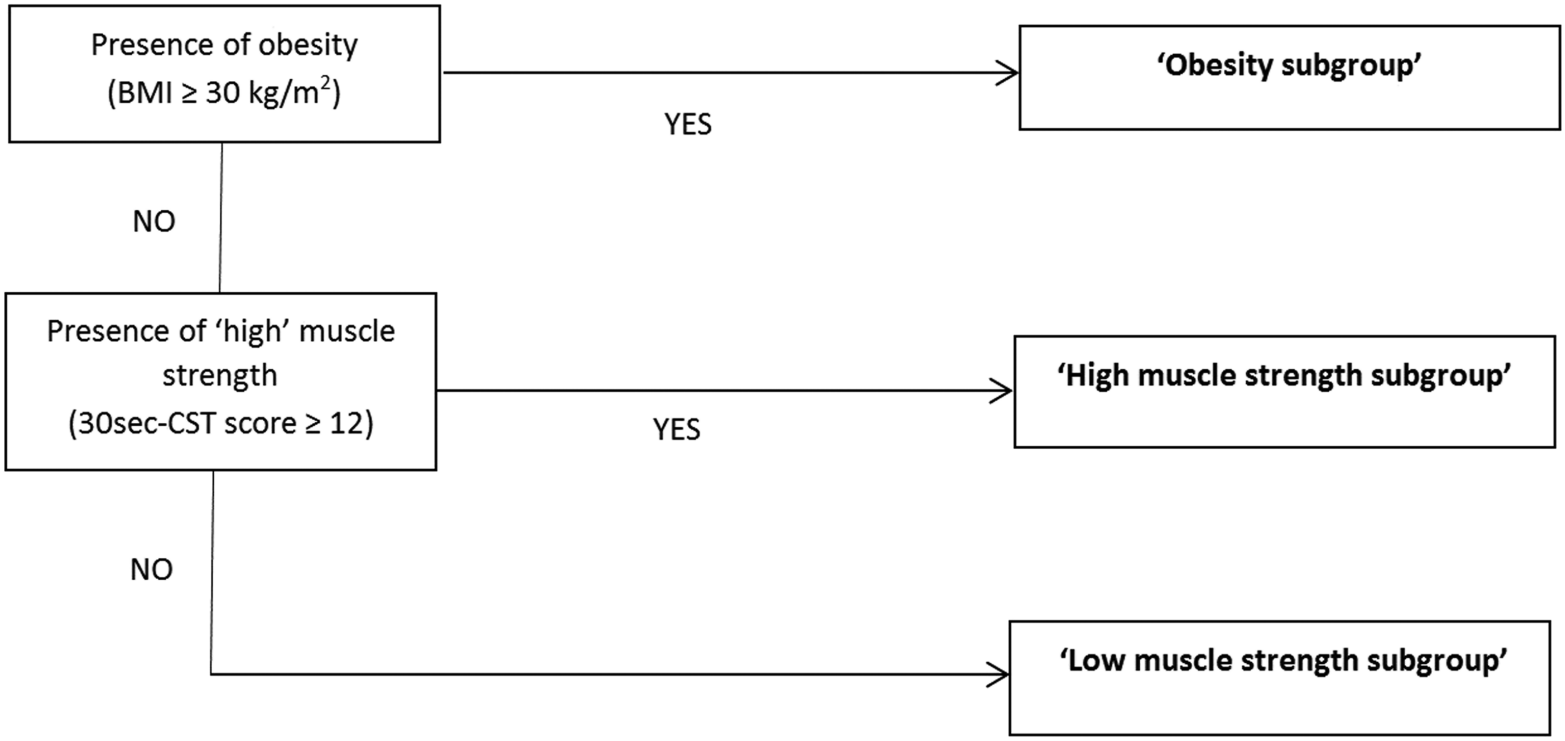
Stratification algorithm in the OCTOPuS‐study

1. Subgroup allocation by PT through a simple, stratification algorithm, based on BMI and upper leg muscle strength (assessed by 30‐s chair stand test; see Figure 2).
2. Subgroup‐specific, protocolized exercise therapy interventions for high muscle strength subgroup, low muscle strength subgroup, and obesity subgroup (see Table 1).

For each of the subgroups, exercise therapy is provided with training parameters as recommended for optimal training effects (ACSM, 2018). The intended training intensity for strengthening exercises is three series of 10 repetitions on 60–80% of the 1‐repetition maximum and for aerobic exercises three intervals of 1 to 2 min on at least 60% of the maximal heart rate (or if possible, three intervals of 30–60 s on 80% of the maximal heart rate). PTs should aim to increase training intensity weekly. The intended training dosage (supervised and home exercises combined) for strengthening exercises is three to 5–8 exercises per day on 2 to 3 days week^−1^; for aerobic exercises, this starts with 10 min day^−1^, increasing to 150 min week^−1^ in 3 to 5 days week^−1^. PTs were provided a large number of (strengthening and aerobic) exercises but were allowed to use other (strengthening and aerobic) exercises as well.

**Table 1:** Description of subgroup‐specific, protocolized exercise therapy interventions

	High muscle strength subgroup	Low muscle strength subgroup	Obesity subgroup
Number of PT sessions	‐ 3 to 4 sessions from PT during 3‐month treatment period	‐ 8 to 12 sessions during 3‐month treatment period	‐ 12 to 18 sessions during 3‐month treatment period
		‐ 1 to 2 additional (“booster”) sessions during 9‐month follow‐up period	
			‐ 2 to 3 additional (“booster”) sessions during 9‐month follow‐up period
	‐ 1 additional (“booster”) session during 9‐month follow‐up period		
Content of PT	‐ Education specifically focusing on self‐management strategies to remain physically active but prevent knee overloading (due to specific physical activities or too many activities), next to information on knee OA disease and symptoms	‐ Education specifically focusing on self‐management strategies to start and maintain a physical active lifestyle, next to information on knee OA disease and symptoms	‐ Education specifically focusing on self‐management strategies to start and maintain a physical active lifestyle but prevent knee overloading (due to overweight) and to lose weight, next to information on knee OA disease and symptoms
		‐ Home exercises targeting muscle strengthening	
		‐ Supervised exercise therapy primarily targeting muscle strengthening, based on our previously tested exercise protocol for knee OA patients (Knoop et al., 2013).	
			‐ Home exercises targeting both muscle strengthening and improving aerobic capacity
			‐ Supervised, adapted exercise therapy targeting both muscle strengthening and aerobic capacity, with exercises adapted to the presence of obesity and with motivational interviewing techniques incorporated, based on our previously tested exercise protocol for knee OA patients with obesity (de Rooij et al., 2017).
	‐ Home exercises to sustain adequate muscle strength and physical active lifestyle		
Number of sessions dietician	n/a	n/a	‐ 3 to 4 sessions during 3‐month treatment period
			‐ 2 to 4 additional (“booster”) sessions during 9‐month follow‐up period
Content of treatment dietician	n/a	n/a	‐ Advising, motivating, and monitoring of healthy diet and active lifestyle, aiming at 10% weight loss in 12 months, according to current guideline (van Binsbergen et al., 2010). In line with this guideline, a personalized advice on healthy diet will be provided and monitored by the dietician (with no specific type of diet or dietary supplements recommended) and facilitated through motivational interviewing techniques for long‐lasting effects
Other			‐ Interprofessional collaboration between PT and dietician during treatment period

Abbreviations: OA, osteoarthritis; PT, physical therapy/physical therapist.

#### 2.6.2 Control intervention

In the control group, PTs are instructed to provide usual best practice, similar to what they provided to knee OA patients before the start of the study (i.e., standard, nonstratified exercise therapy following the guideline, Peter, Jansen, Hurkmans, Bloo, Dekker et al., 2011). Patients receiving the control intervention are thereby treated similarly as all knee OA patients treated in usual care, which has been reported to consist of on average 17 sessions in the Netherlands (Leemrijse et al., 2016). Patients are allowed to receive additional usual care from other clinician and health professionals, which will be monitored by follow‐up questionnaires.

### 2.7 Measurements

We perform measurements at baseline (t0), 3 months (t3; primary end point), 6 months (t6), and 12 months (t12), with an additional cost measurement at 9 months (t9), to avoid “recall bias” (Goossens, Rutten‐van Mölken, Vlaeyen, & van der Linden, 2000). The physical tests will be performed at t0 (supervised by PT), t3 (by PT), and t12 (by researcher). At baseline, we will collect general baseline characteristics not only from patients but also from PTs, in order to check similarity between arms. In Table 2, an overview is provided of all (outcome) measures and tests for the different time‐points.

**Table 2:** Overview of measurements

Measures	t0	t3	t6	t9	t12
Effectiveness evaluation: primary outcome measures					
Knee pain severity, assessed by 1‐item NRS knee pain (on average during walking in the past week; score between 0 and 10; 0 = no pain; 10 = worst pain imaginable; McCaffery & Pasero, 2001)	X	X	X		X
Physical functioning, assessed by 17‐item subscale function in daily living (ADL) of the Dutch translation of the Knee Injury and Osteoarthritis Outcome Score (KOOS) questionnaire (score between 0 and 100; 0 = maximal problems; 100 = no problem, in the past week; de Groot et al., 2008; Roos, Roos, Lohmander, Ekdahl, & Beynnon, 1998)	X	X	X		X
Effectiveness evaluation: secondary outcome measures					
Global perceived effect (GPE; 1 item). This item measures the patient's subjective global improvement using a 7‐point scale (score ranging from 1 = *completely recovered* to 7 = *worse than ever*)		X	X		X
Physical functioning, assessed by the 7‐item short version of the Dutch translation of the Knee Injury and Osteoarthritis Outcome Score (KOOS) questionnaire (score between 0 and 100; 0 = maximal problems; 100 = no problem, in the past week; de Groot et al., 2008; Roos et al., 1998)	X	X	X		X

Pain interference, assessed by the 4‐item short version of the Patient Reported Outcomes Measurement Information System (PROMIS)—Pain interference (Khanna et al., 2011)	X	X	X		X
Fatigue, assessed by the 4‐item short version of the Patient Reported Outcomes Measurement Information System (PROMIS)—Fatigue (Khanna et al., 2011)	X	X	X		X
Patient‐reported knee instability, consisting of 2 items (frequency of episodes of knee instability in the past 3 months and impact of knee instability on daily living; Knoop et al., 2012)	X	X	X		X
Upper leg muscle strength, assessed by the 30‐s chair stand test (30‐s CST). This performance test measures the maximum number of chair stand repetitions possible in a 30‐s period and is considered a measure of lower body strength as well as a performance‐based measure for physical functioning (Dobson et al., 2012)	X^a^	X^a^			X^b^
Body mass index (BMI): body weight (in kilograms) divided by squared body length (in metres)	X^a^	X^a^			X^b^
Waist circumference: distance around the abdomen in the horizontal plane midway between the lowest rib and the iliac crest (in centimetres; van Binsbergen et al., 2010)	X^a^	X^a^			X^b^

Economic evaluation					
Intervention‐related costs, assessed using a bottom‐up microcosting approach in which detailed data will be collected regarding the number and kinds of resources used as well as their respective unit prices (Goossens et al., 2000)	X	X	X	X	X
Other health care costs, including primary and secondary costs and prescribed medication, assessed using a cost questionnaire and valued in accordance with the Dutch Manual of Costing (Goossens et al., 2000)	X	X	X	X	X
Patient costs, including over‐the‐counter medication, informal care, and unpaid productivity costs, assessed using a cost questionnaire and valued using a recommended Dutch shadow price (Goossens et al., 2000)	X	X	X	X	X
Work‐related productivity costs, including patient‐reported productivity (work ability), and absenteeism (i.e., costs of being absent from work) and presenteeism costs (i.e., costs of being less productive while being at work), with costs valued using gender‐specific price weights (Goossens et al., 2000)	X^c^	X^c^	X^c^	X^c^	X^c^

Quality‐adjusted life years (QALYs), assessed by the EuroQol‐5D‐5L (EQ‐5D‐5L; 5 items), in which the patient can self‐rate their level of severity on five domains (mobility, self‐care, daily activities, pain/discomfort, and anxiety/depression; Kind, 1996). For the economic evaluation, the EQ‐5D‐5L health states will be converted into utility scores ranging from 0 (*death*) to 1 (*optimal health*) using the Dutch tariff (Hakkaart‐van Roijen, Van der Linden, Bouwmans, Kanters, & Tan, 2015; Kind, 1996). QALYs are calculated using the area under the curve approach	X	X	X		X
Process evaluation					
Number of sessions, registered by PT/dietician		X^d^			
Type of interventions per session, registered by PT/dietician		X^d^			
Training intensity (exertion) per session, perceived by patient (Borg scale, Borg, 1970) and estimated by PT (i.e., higher/similar/lower than previous sessions), both registered by PT		X^d^			
Adverse events per session, registered by PT/dietician		X^d^			
Adherence to home exercises (number of days performing exercises and number of exercises), reported by patient		X	X		X
Adherence to (moderate‐intensity and high‐intensity) physical activities (number of days week^−1^ and number of min/day performing physical activities per week and type of activity; separately for moderate‐intensity and high‐intensity activities), reported by patient		X	X		X

Qualitative information (barriers and facilitators) on treatment content, fidelity, and adherence, collected through individual, semistructured interviews using “topic lists” in a random sample of patients from both treatment arms (*n* = 20), PTs from both treatment arms (*n* = 10), and dieticians (*n* = 5)					X
General patient characteristics					
Age	X				
Gender	X				
Symptomatic knee joint (left/right/both)	X				
Duration of knee symptoms	X				
History of knee surgery	X				
Co‐morbidities	X				
General PT characteristics					
Age	X				
Gender	X				
Amount of years working as PT	X				
Number of knee OA patients treated per month	X				
Member of OA network	X				
Specializations	X				
OA‐related courses	X				

Abbreviations: NRS, Numeric Rating Scale; OA, osteoarthritis; PT, physical therapist.

a Assessed by PT.

b Assessed by researcher.

c Only for patients with paid job.

d Continuously registered by PT and dietician (for each session).

### 2.8 Sample size

According to the Cochrane review from Fransen et al. (2015), the average (between‐group) effect size (ES) of usual exercise therapy in knee OA on knee pain is 0.49, corresponding with a between‐group difference in mean improvement of 1.2 points (on 0–10 scale). In an RCT, comparable with our proposed study but in low back pain (Hill et al., 2011), stratified exercise therapy by a PT resulted in a (between‐group) ES of 0.3, compared with usual exercise therapy, corresponding with a between‐group difference in mean improvement of 0.5 points (on 0–10 scale). Based on a mean improvement 1.2 points (on 0–10 scale) for usual exercise therapy, our experimental intervention should result in a mean improvement of 1.7 points (on 0–10 scale). As this is lower than the mean improvement of 2.1 points found in our uncontrolled pilot study (*n* = 50; Knoop et al., 2019), this effect is highly plausible. With an expected between‐group difference in mean improvement of 0.5 points (on 0–10 scale), an estimated standard deviation of 1.4, α = .05 (two‐sided testing), power = 90%, design effect of 1.05 (with an estimated mean number of patients per cluster [i.e., PT practice] of 6), and 15% dropout rate, 408 patients (204 per group) will be needed.

### 2.9 Analysis

#### 2.9.1 Effectiveness evaluation

All patients included in the study will be analysed, with missing data from withdrawals handled by maximum likelihood estimation (“dealing with missing values”). Statistical significance will be accepted at *p* values of less than .05 (two‐sided testing). Baseline patient and PT characteristics in both study groups will be presented using descriptive statistics (mean/standard deviation, median/range, or proportions) to check similarity between arms. Descriptive statistics comparing both study groups will also be calculated for other study parameters (e.g., costs and process parameters). The primary analysis will be an intention‐to‐treat, multilevel, regression analysis comparing stratified versus usual care for the primary outcome measures, with 3‐month follow‐up as a primary time‐point and 6‐ and 12‐month follow‐up as secondary time‐points. In this analysis, we will take into account the level of PT practice, level of patient, and level of time‐point. In addition, we will perform a post hoc analysis with an additional level of PT, with the appropriateness of this additional level tested using the −log 2 likelihood test. Furthermore, we will perform a post hoc analysis comparing stratified versus usual care but now separately for each of the three subgroups. Only if deemed necessary, the analysis should be adjusted for important prognostic characteristics that potentially confound the treatment effects (e.g., pain severity, duration of symptoms, upper leg muscle strength, and BMI) or for important baseline patient characteristics (e.g., outcome measure) or PT characteristics that substantially differ between the two arms. We will perform similar analyses for secondary outcomes measures, except for GPE, which is analysed as a dichotomous variable (“completely recovered” and “much recovered” vs. all other responses), using logistic multilevel analysis.

#### 2.9.2 Economic evaluation

We will perform an economic evaluation from a societal and a health care perspective for quality‐adjusted life years, knee pain, and physical function. All patients included in the study will be analysed, with missing data handled by using multiple imputation by chained equations. Costs and effect differences will be estimated using seemingly unrelated regression analyses, in which their possible correlation can be accounted for. The 95% confidence intervals surrounding the cost differences will be estimated using bias‐corrected and accelerated bootstrapping. Subsequently, incremental cost‐effectiveness ratios will be estimated by dividing the differences in costs by the differences in effects. Uncertainty surrounding the incremental cost‐effectiveness ratios will be graphically illustrated by plotting BCA‐bootstrapped cost‐effect pairs on cost‐effectiveness planes. Also, cost‐effectiveness acceptability curves will be constructed to provide an indication of the probability of stratified exercise therapy being cost‐effective with usual care at different values of willingness to pay (Drummond, Sculpher, & Torrance, 2006).

#### 2.9.3 Process evaluation

We will analyse all process parameters descriptively, in which treatment arms will be compared. In addition, qualitative information from the interviews will be analysed descriptively.

### 2.10 Timelines

Recruitment and training of PTs occurred between June and December 2018. Ethics approval was obtained at the VU University Medical Center in January 2019. Start of the trial and patient inclusion occurred after this approval.

The anticipated timeline for the CRCT is as follows:

January 2019: Start of inclusion and data collection
February 2020: Inclusion completed
May 2020: All participants completed t3 (immediate post‐treatment)
February 2021: End of data collection: All participants completed t12

## 3 DISCUSSION

OA is one of the most disabling diseases, costing globally more than 150 billion dollar each year (Ma, Chan, & Carruthers, 2014). Exercise therapy is recommended as a first step treatment option for patients with knee OA in all relevant clinical guidelines. We hypothesize that with our model of stratified care, PTs will be able to optimize their knee OA treatment in a cost‐effective manner. In addition, dieticians may also be able to optimize their treatment in obese patients with knee OA by using our model and collaborating more strongly with the PT. The stratified care model, tested in the undergoing trial, was developed on the basis of our previous research on optimization of exercise therapy effects (de Rooij et al., 2017; Edelaar et al., 2017; Knoop et al., 2013; Knoop et al., 2014; Knoop et al., 2015) and clinical phenotypes (Knoop et al., 2011; van der Esch et al., 2015) and was found to be feasible in a primary care setting (Knoop et al., 2019). Phenotyping and stratified care are considered the “holy grail” in medical research (Foster, Hill, O'Sullivan, & Hancock, 2013). For stratified care development, different approaches can be used: a prognostic risk approach, a responsiveness to treatment approach, or a aetiological/mechanistic approach (Foster et al., 2013). In contrast to the well‐known model of stratified care in low back pain that was based on the first approach (using the STarT Back Screening Tool, Hill et al., 2011), our stratified care model had been developed from a clinical perspective using a combination of the second and third approach. It has thereby the potential to optimize (cost‐)effectiveness of exercise therapy for a very large and strongly increasing patient group. Even more important, due to the simplicity of the stratification algorithm, our model, if proven to be (cost‐)effective, can easily be implemented in daily practice and quickly adopted in current guidelines, similar as for the STarT Back Screening Tool (de Campos, 2017).

Our study design has several other strengths. First, this trial will be conducted in a primary care setting in “real‐world” patients in a large number of patients and PTs, so our findings can be expected to be representative for daily practice. Second, our intervention has already been found to be feasible in primary care with promising results on outcome measures but had been further improved on the basis of the study findings from our pilot study (Knoop et al., 2019). Therefore, it is unlikely that we will face feasibility issues during our trial. Third, our sample size has been calculated to detect minimal clinically important differences between groups and also enable us to perform explorative, secondary analyses to compare both interventions within each subgroup. Fourth, we added a dietary intervention to our subgroup‐specific exercise therapy intervention for the obesity subgroup. This interdisciplinary approach is not provided in usual care and can be expected to optimize current treatment effects in this complex and increasing patient group. Fifth, our outcome measures cover a broad spectrum of clinical and economic constructs, whereas we also collect detailed (quantitative and qualitative) information on treatment content, fidelity, and adherence. Thereby, the OCTOPuS study will gain new insights how to further optimize exercise therapy for patients with knee OA.

## 4 IMPLICATIONS FOR PHYSIOTHERAPY PRACTICE

Exercise therapy is recommended as a first step treatment option for patients with knee OA in all relevant clinical guidelines, but current clinical effects are modest and should be further optimized. A stratified approach to exercise therapy, whereby patients are allocated to homogeneous subgroups and receive a stratified, subgroup‐specific intervention, can be expected to optimize current clinical effects. This new approach, which was found to be feasible with promising effects in a pilot study, is currently being tested for its effectiveness and cost‐effectiveness compared with usual best practice by PTs in in a CRCT (OCTOPuS study). If proven to be (cost‐)effective, this intervention can easily be implemented in daily practice and quickly adopted in current guidelines.

## ETHICS APPROVAL AND CONSENT TO PARTICIPATE

This study has been approved by the Medical Ethical Committee of the VU University Medical Centre (2018.563). We conduct this study in agreement with the Declaration of Helsinki (2013), in accordance with the Dutch Medical Research Involving Human Subjects Act (WMO) and the General Data Protection Regulation (in Dutch: Algemene Verordening Gegevensbescherming, AVG). We obtain written informed consent from each subject, after the information letter has been provided. The researchers make sure that the participants are given complete, adequate, written, and oral information regarding the nature, aims, possible risks, and benefits of the study. We explain to the participants that they are free to interrupt their participation in the study at any moment without any consequence and that they are able to receive a digital copy of their personal data. Participants should keep a copy of the information sheet and informed consent form. An independent expert has been appointed to provide subjects the opportunity to ask questions about the study to someone not related to the study.

## AVAILABILITY OF DATA AND MATERIALS

The datasets generated and/or analysed during the current study will be available in the Dataverse repository (https://dataverse.nl/dataverse/BETA). The research protocol is accessible at the Netherlands National Trial Register (https://www.trialregister.nl/trial/7463) and Open Science Framework (https://osf.io/x3p94/).

## TRIAL REGISTRATION

The Netherlands National Trial Register (NTR): NL7463 (date of registration: January 8, 2019).

## AUTHORSHIP

The authors declare that they have no competing interests and that they qualify for authorship according to the following criteria:

1. have made substantial contributions to conception and design, or acquisition of data, or analysis and interpretation of data;
2. been involved in drafting the manuscript or revising it critically for important intellectual content;
3. given final approval of the version to be published. Each author should have participated sufficiently in the work to take public responsibility for appropriate portions of the content; and
4. agreed to be accountable for all aspects of the work in ensuring that questions related to the accuracy or integrity of any part of the work are appropriately investigated and resolved.
